# Distribution of pediatric keratoconus by different age and gender groups

**DOI:** 10.3389/fped.2022.937246

**Published:** 2022-07-18

**Authors:** Kaili Yang, Yuwei Gu, Liyan Xu, Qi Fan, Meng Zhu, Qing Wang, Shanshan Yin, Bo Zhang, Chenjiu Pang, Shengwei Ren

**Affiliations:** Henan Provincial People’s Hospital, Henan Eye Hospital, Henan Eye Institute, People’s Hospital of Zhengzhou University, Henan University People’s Hospital, Zhengzhou, China

**Keywords:** pediatric keratoconus, keratoconus severity, age distribution, gender distribution, China

## Abstract

**Purpose:**

To evaluate the distribution of pediatric keratoconus patients and the disease severity based on different age and gender groups in China.

**Materials and methods:**

A total of 446 keratoconus eyes in 266 pediatric patients from January 2019 to January 2022 were included in the cross-sectional study. The clinical findings and severity of keratoconus were recorded and Kruskal–Wallis test, chi-squared test, and Cochran-Mantel-Haenszel (CMH) test were used to compare the parameters between different gender and age groups.

**Results:**

The male/female ratio was 353/93 (3.8:1), and the median age was 16 years (range: 6–17 years). Male patients were statistically younger than female patients (*P* = 0.041). The male/female ratio decreased with age (*P* for trend = 0.011). The distribution of the topographic keratoconus classification (TKC) stage was significantly different between gender and age groups (all *P* < 0.05). Male patients had a higher ratio of advanced keratoconus eyes (TKC ≥ 3) than female patients (*P* < 0.001), and CMH analysis indicated that being a male was a risk factor for advanced keratoconus after controlling for age (odds ratio: 2.581, *P* < 0.001).

**Conclusion:**

Male keratoconus patients were younger, with a higher ratio of advanced keratoconus than female patients in the Chinese pediatric patients evaluated. Multicenter studies with larger sample sizes are necessary in the future.

## Introduction

Keratoconus is a corneal disorder usually characterized by corneal thinning, vision deterioration, and irregular astigmatism (1). Its prevalence has been reported at approximately 1.38/1000 (2, 3). Keratoconus poses a burden on the society as it affects the quality of life, affecting social and educational developments (4). Keratoconus is typically manifested at puberty and progresses until the third or fourth decade of life (1, 5). The distribution of keratoconus severity varies in different age groups, (6–8) and males account for a higher proportion of keratoconus cases than females according to previous studies (8, 9). However, subgroup analysis combining gender and age is more helpful to elucidate the characteristics of keratoconus in detail, which is limited in application. Meanwhile, previous studies on the characteristics of keratoconus have mostly focused on adult keratoconus or a combination of adult and pediatric patients, with limited focus on pediatric keratoconus (8–10).

As a key keratoconus population, pediatric keratoconus refers to patients <18 years of age (11–13). Although pediatric keratoconus shares most of the common signs and symptoms of adult keratoconus, the time of presentation, disease progression rate, and treatment protocols are different between pediatric and adult keratoconus (14–16). It has been reported that nearly half of the pediatric keratoconus cases present with progressive disease, which is more aggressive than in adult patients (13, 17). Therefore, the distribution of pediatric keratoconus, which is in the early stages of the disease, has been reported inadequately but is of great value, especially in different age and gender groups (18, 19). A recent multicenter study of pediatric keratoconus showed that the age was not different between male and female patients, with female patients exhibiting a higher topographic keratoconus classification (TKC) value than male patients (18). The multicenter study patients were from Spain, the United Kingdom, and Latvia, with all in the European region, and the results were different from those reported by Mahmoud et al. (19) who found that the age and keratoconus severity were not different between male and female patients in Egypt pediatric keratoconus. Considering the discrepancies in keratoconus characteristics in different geographic locations and the relatively high prevalence of pediatric keratoconus in China, (11, 20) the current study aimed to evaluate the distribution of Chinese pediatric keratoconus patients in terms of different gender and age groups, and to provide a reference for clinical management of pediatric keratoconus.

## Materials and methods

### Study subjects

**FIGURE 1:**
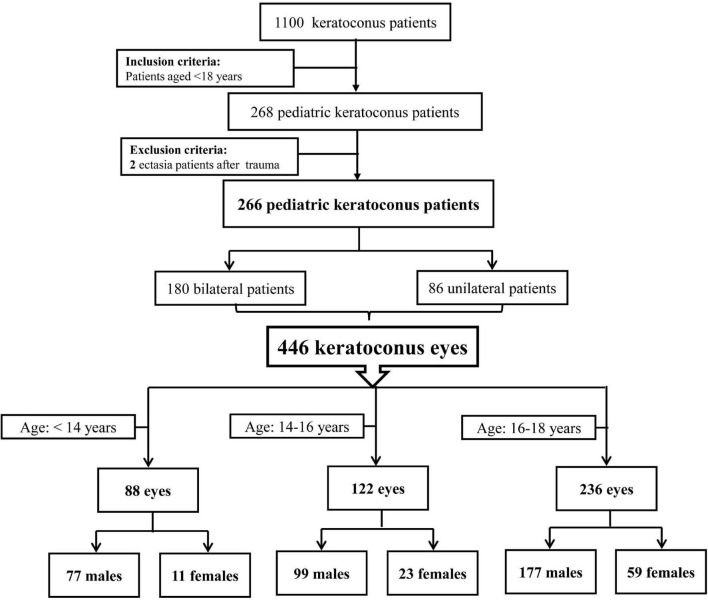
Flow diagram of study subjects.

The pediatric keratoconus patients <18 years of age were consecutively enrolled in this cross-sectional study from January 2019 to January 2022 in Henan Eye Hospital and Henan Eye Institute. Keratoconus was diagnosed according to the following criteria: (21, 22) an asymmetric bowtie pattern with or without skewed axes revealed and Belin Ambrosio enhanced ectasia total deviation index (BAD) value>2.6 by corneal topography, keratoconus signs detected by the slit-lamp examination (localized stromal thinning, Vogt’s striae, Fleischer’s ring, conical protrusion, or anterior stromal scar). Patients with a history of ectasia after trauma were excluded. The detailed inclusion and exclusion criteria of study subjects are presented in Figure 1. Finally, 446 keratoconus eyes in 266 pediatric patients were included in the current analysis. This study was conducted according to the Declaration of Helsinki guidelines and approved by the Institutional Review Board of Henan Eye Hospital [ethical approval number: HNEECKY-2019 (5)]. Informed consent was obtained from the legal guardians of pediatric patients.

### Clinical examination data

All the patients underwent the following clinical examinations: spherical equivalent, cylinder, and corrected distance visual acuity (CDVA), slit-lamp examination, intraocular pressure (IOP), axial length measurement, and corneal topography and tomography analysis. A standard logarithmic visual acuity chart was used to obtain visual acuity values, and CDVA was converted to the logarithm of the minimum angle of resolution (logMAR) unit and used for further analyses. Corneal topography and tomography were obtained through Pentacam HR (Oculus, Wetzlar, Germany), which uses the Scheimpflug imaging technique to present indices with acceptable accuracy and repeatability (23). The following parameters were evaluated in the current study: corneal thickness (apex corneal thickness: ACT; pupil corneal thickness: PCT; thinnest corneal thickness: TCT), keratometry (flat keratometry: K1; steep keratometry: K2; mean keratometry: Km; maximum keratometry: Kmax), inferior superior value (IS-value), BAD, and TKC (a classification system of keratoconus as provided by Pentacam HR) (24).

The clinical examinations were conducted when the patients were first referred to our center. Patients wearing RGP at their first visit were asked to stop wearing it for 2 weeks and were examined again. The measurements were conducted by experienced operators between 9:00 and 17:00 to ensure that the results could be used for analyses.

### Statistical analysis

The median and interquartile range (P25 and P75) were applied to describe qualitative data, and proportion was used to show quantitative data. The distribution differences of parameters in gender and age groups (group 1: < 14 years, group 2: 14–16, and group 3: 16–18) were compared through the Kruskal–Wallis test or chi-squared test. To better explore the distribution of keratoconus severity, the TKC stage was divided into two groups (mild and moderate keratoconus: TKC <3 and advanced keratoconus: TKC ≥ 3) (25). Cochran-Mantel-Haenszel (CMH) chi-squared test with age adjustment was used to investigate the association between gender and keratoconus severity. Chi-squared test for trend test was used to test the linear trend of the keratoconus severity in age and gender groups. *P* < 0.05 (two-tailed) was considered a statistically significant difference.

## Results

### Characters of total pediatric keratoconus eyes

**FIGURE 2:**
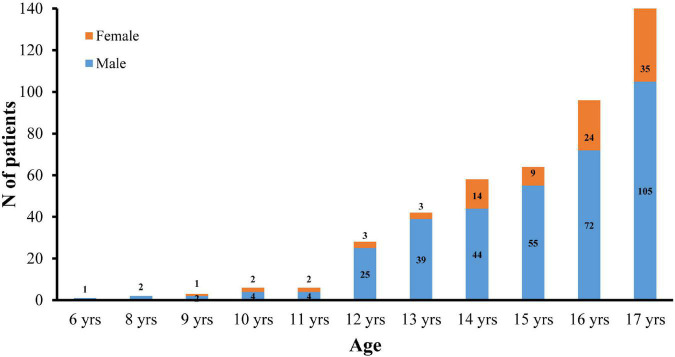
Gender distribution of pediatric KC patients in different age groups.

Table 1 presents the characteristics of 446 pediatric keratoconus eyes. The male/female ratio was 353/93 (3.8:1), and the median age was 16 years (range: 6–17 years). There were 88 eyes (19.7%) in patients <14 years of age. Figure 2 presents the distribution of male and female patients in different age groups. In addition, the median CDVA (logMAR) for current pediatric keratoconus eyes was 0.30 (0.15, 0.52). The median TCT was 461 μm with an interquartile range of 70 μm. Median Kmax and BAD of the eyes were 57.31 (50.68, 69.50) D and 7.55 (4.22, 13.52), respectively.

**TABLE 1:** The data characters of total pediatric keratoconus eyes.

Parameter	Median (P25, P75)/N (%)
Number	446
Enrollment age (years)	16(14,17)
**Age group**	
<14 years	88 (19.73)
14–16 years	122 (27.35)
16–18 years	236 (52.91)
**Gender**	
Male	353 (79.15)
Female	93 (20.85)
CDVA (logMAR)	0.30(0.15,0.52)
IOP (mmHg)	12.5(11.0,14.5)
Axial length (mm)	24.93(24.21,25.81)
ACT (μm)	471(435,503)
PCT (μm)	482(451,510)
TCT (μm)	461(422,492)
K1 (D)	46.4(43.6,53.1)
K2 (D)	50.9(46.7,58.3)
Km (D)	48.8(45.3,55.7)
Kmax (D)	57.31(50.68,69.50)
IS-value	4.41(2.12,6.85)
BAD	7.55(4.22,13.52)
**TKC stage**	
0	35 (7.85)
1	49 (10.99)
1–2	33 (7.40)
2	99 (22.20)
2–3	29 (6.50)
3	61 (13.68)
3–4	75 (16.82)
4	65 (14.57)

IQR, interquartile range; CDVA, corrected distance visual acuity; IOP, intraocular pressure; ACT, apex corneal thickness; PCT, pupil corneal thickness; TCT, thinnest corneal thickness; K1, flat keratometry; K2, steep keratometry; Km, mean keratometry; Kmax, maximum keratometry; IS-value, inferior superior value; BAD, Belin Ambrósio display; TKC, topographic keratoconus classification.

### Distribution of keratoconus by gender group

Table 2 presents the data comparison based on gender groups. Males were significantly younger than females (mean ranks: 217.26 vs. 247.18, *P* = 0.041). The median CDVA (logMAR), Kmax, and IS-value in males were higher than in females, while the IOP in males was lower than in females (all *P* < 0.05). The distribution of the TKC stage was significantly different between males and females (*P* = 0.007, Table 2).

**TABLE 2:** Distribution of keratoconus by gender group.

Parameter, Median (P25, P75)/N (%)	Male (*N* = 353)	Female (*N* = 93)	*P*
Enrollment age (years)	16(14,17)	16(14,17)	0.041
CDVA (logMAR)	0.40(0.15,0.70)	0.30(0.10,0.52)	0.045
IOP (mmHg)	12.3(10.5,14.0)	13.5(11.5,15.0)	0.002
Axial length (mm)	24.96(24.33,25.81)	24.63(23.79,25.80)	0.094
ACT (μm)	468(434,502)	480(443,508)	0.126
PCT (μm)	480(449,509)	487(457,512)	0.202
TCT (μm)	456(419,492)	468(436,495)	0.052
K1 (D)	46.9(43.5,54.4)	45.7(44.1,50.2)	0.268
K2 (D)	51.3(46.7,59.6)	49.6(46.7,54.4)	0.111
Km (D)	49.3(45.3,56.7)	47.4(45.2,52.4)	0.217
Kmax (D)	58.41(50.91,72.18)	55.04(50.01,60.48)	0.016
IS-Value	4.85(2.17,7.16)	3.46(1.91,5.52)	0.014
BAD	7.72(4.27,14.50)	7.13(4.04,10.79)	0.094
TKC stage			0.007
0	28 (7.93)	7 (7.53)	
1	37 (10.48)	12 (12.90)	
1–2	23 (6.52)	10 (10.75)	
2	71 (20.11)	28 (30.11)	
2–3	19 (5.38)	10 (10.75)	
3	55 (15.58)	6 (6.45)	
3–4	68 (19.26)	7 (7.53)	
4	52 (14.73)	13 (13.98)	

IQR, interquartile range; CDVA, corrected distance visual acuity; IOP, intraocular pressure; ACT, apex corneal thickness; PCT, pupil corneal thickness; TCT, thinnest corneal thickness; K1, flat keratometry; K2, steep keratometry; Km, mean keratometry; Kmax, maximum keratometry; IS-value, inferior superior value; BAD, Belin Ambrósio display; TKC, topographic keratoconus classification.

### Distribution of keratoconus by age groups

Table 3 presents the data comparison between age groups. The distributions of gender, axial length, and TKC stage were significantly different between the three age groups (all *P* < 0.05). In addition, the male/female ratio decreased with age (*P*_for trend_ = 0.011).

**TABLE 3:** Distribution of keratoconus by age groups.

Parameter, Median (P25, P75)/N (%)	<14 years (*N* = 88)	14–16 years (*N* = 122)	16–18 years (*N* = 236)	*P*
Gender				0.039
Male	77 (87.50)	99 (81.15)	177 (75.00)	
Female	11 (12.50)	23 (18.85)	59 (25.00)	
CDVA (logMAR)	0.30(0.10,0.52)	0.40(0.15,0.70)	0.30(0.15,0.52)	0.528
IOP (mmHg)	12(11,14.5)	13(10.5,15)	12.5(11,14)	0.825
Axial length (mm)	24.38(23.34,25.44)	24.92(24.38,25.91)	25.15(24.36,25.92)	<0.001
ACT (μm)	471(445.75,501.5)	478(435,505)	466(431,502.75)	0.256
PCT (μm)	483(457,508.5)	487(453.5,515.5)	479.5(448,509)	0.527
TCT (μm)	461(430.75,491)	463(420,492.75)	457.5(415.75,493)	0.372
K1 (D)	45.5(43.6,51.1)	47.2(43.53,54.8)	46.15(43.7,53.33)	0.226
K2 (D)	50.2(47.5,57.5)	51.6(46.73,60.18)	50.4(46.6,58.2)	0.860
Km (D)	47.9(45.4,54.1)	49.45(45,56.78)	48.6(45.2,55.7)	0.522
Kmax (D)	55.39(51.75,70.06)	58.27(50.82,70.06)	57.57(50.04,68.24)	0.592
IS-Value	4.26(2.37,6.50)	4.62(2.10,6.87)	4.73(2.05,7.04)	0.796
BAD	7.67(4.67,13.05)	7.45(3.91,13.22)	7.78(4.25,13.88)	0.812
TKC stage				0.047
0	10 (11.36)	9 (7.38)	16 (6.78)	
1	3 (3.41)	13 (10.66)	33 (13.98)	
1–2	7 (7.95)	5 (4.10)	21 (8.90)	
2	24 (27.27)	31 (25.41)	44 (18.64)	
2–3	6 (6.82)	7 (5.74)	16 (6.78)	
3	15 (17.05)	13 (10.66)	33 (13.98)	
3–4	18 (20.45)	24 (19.67)	33 (13.98)	
4	5 (5.68)	20 (16.39)	40 (16.95)	

IQR, interquartile range; years, years; CDVA, corrected distance visual acuity; IOP, intraocular pressure; ACT, apex corneal thickness; PCT, pupil corneal thickness; TCT, thinnest corneal thickness; K1, flat keratometry; K2, steep keratometry; Km, mean keratometry; Kmax, maximum keratometry; IS-value, inferior superior value; BAD, Belin Ambrósio display; TKC, topographic keratoconus classification.

### Keratoconus severity according to different gender and age groups

When further sub-classified based on the TKC stage, there were 201 advanced keratoconus eyes (45.07%) in the current study (Table 4). Males had a higher ratio of advanced keratoconus eyes than females (*P* < 0.001). No significant difference was found between age groups and keratoconus severity (*P* = 0.877). Further CMH analysis indicated that male had a high ratio of advanced keratoconus in the 14–16 and 16–18 year age groups. Compared to being a female, being a male was a risk factor for advanced keratoconus after controlling for age (odds ratio: 2.581, 95% confidence interval: 1.562–4.266, *P* < 0.001).

**TABLE 4:** Distribution of keratoconus severity according to gender and age groups.

Type, N (%)	TKC < 3 (*N* = 245)	TKC ≥ 3 (*N* = 201)	χ ^2^	*P*	*P*_for trend_
**Gender group**			13.895	<0.001	<0.001
Male	178 (50.42)	175 (49.58)			
Female	67 (72.04)	26 (27.96)			
**Age group**			0.263	0.877	0.875
<14 years	50 (56.82)	38 (43.18)			
14–16 years	65 (53.28)	57 (46.72)			
16–18 years	130 (55.08)	106 (44.92)			
**Gender combined age group**					
<14 years			3.202	0.074	0.075
Male	41 (53.25)	36 (46.75)			
Female	9 (81.82)	2 (18.18)			
14–16 years			7.106	0.008	0.008
Male	47 (47.47)	52 (52.53)			
Female	18 (78.26)	5 (21.74)			
16–18 years			5.138	0.023	0.024
Male	90 (50.85)	87 (49.15)			
Female	40 (67.80)	19 (32.20)			

TKC, topographic keratoconus classification.

## Discussion

Pediatric keratoconus is an important condition, and studying pediatric keratoconus is essential for improving its clinical management (11, 12). The present study showed that the male/female ratio was 3.8:1, and the ratio decreased with age. In addition, male patients were significantly younger than female ones, and being a male was a risk factor for advanced keratoconus.

Pediatric keratoconus tends to present more aggressively compared to adult patients (12, 16). Patients with an early onset age appear to have a more severe form of keratoconus, with an increased likelihood of corneal opacities and the subsequent need for keratoplasty (11, 16, 26). In addition, the diagnosis of pediatric keratoconus is often delayed because of few functional complaints and unspecified characteristics (13, 15, 17). Poor vision in children can affect their social and educational development and quality of life (19). Thus, early detection of pediatric keratoconus allows the early use of visual aids and further provides an opportunity to reduce the likelihood of progression, which is of great significance to the future of children and the development of society (13, 14).

A gender-related difference is believed to play an important role in changes in corneal tissue structures (27–30). Male patients account for a higher proportion than female patients based on previous investigations of adult keratoconus (7, 9, 10, 31, 32). A high male/female ratio still exists for pediatric keratoconus. In the current study, the male/female ratio was 79:21, consistent with previous studies by Rocha-de-Lossada et al. (male/female ratio: 75:25) and Gupta et al. (male/female ratio: 74:26) (17, 18). The difference between male and female proportions in keratoconus may be attributed to the level of sex hormones (33). Males have higher levels of androgens, while females have a higher estrogen level (34, 35). The cornea expresses multiple receptors and enzymes for sex hormone metabolism and cellular activities, which further bind to sex hormones, leading to hormonal level changes and further causing other anatomic and physiological differences between males and females (9, 30, 36). In addition, the male/female ratio decreased with age in the current analysis. It might be explained that males and females have different social and cultural roles, expectations, and constraints by virtue of their gender, which cause gender differences in the diagnosis of keratoconus patients (8, 9).

The present study showed that the male patients were younger than the female patients. Further analysis revealed that CDVA (logMAR), Kmax, and IS-value in male patients were higher than in female patients, and the distribution of the TKC stage was significantly different between males and females. These findings indicated an earlier onset and more advanced condition in males than in females, which does not coincide with previous pediatric studies (18, 19). A multicenter study on pediatric patients from Spain, the United Kingdom, and Latvia showed higher values of Kmax, BAD, and TKC in females than in males (18). Mahmoud et al. (19) reported that the age and keratoconus severity were not different between males and females in Egyptian patients with pediatric keratoconus. The sample size, ethnicity, and geographic location could explain the discrepancies in pediatric keratoconus (9). In addition, the current analysis after controlling age showed that males were significantly more likely than females to have advanced keratoconus, suggesting a more rapid disease course in males than in females with pediatric keratoconus. The phenomenon can be explained because at the first diagnosis of keratoconus in children, there is a short interval between noticeable symptoms and the development of advanced symptoms (18). The gender-related differences should receive more attention in the clinical diagnosis and management of pediatric keratoconus. Meanwhile, the causes of gender-related differences in pediatric keratoconus are not well understood, and further studies are necessary to explore the potential mechanisms.

It was proposed that keratoconus starts during adolescence, and the disease progression slows down with age (8, 13, 18). Rocha-de-Lossada et al. (18) found the mean TKC value was lower in adolescents ≤14 years of age than that in those >14. However, no significant difference in keratoconus clinical findings was found in the reports of Mahmoud et al. (19) similar to the present series. The distribution of pediatric keratoconus in different age groups can possibly be attributed to the effects of geographic location, ethnicity, sex hormones, corneal viscoelasticity, and corneal stiffness (34, 37, 38). Among sex hormones, two major classes are androgens and estrogens that peak after puberty in both sexes and then decline with age (34, 35). Kotecha et al. (37) reported a negative correlation between corneal viscoelastic properties and advancing age, and the progressive alteration of the keratoconic corneal shape may be caused by elastic deformation (38). Several experimental studies have shown age-related changes in corneal collagen fibril properties, contributing to an increased corneal stiffness with age (39, 40). Previous studies proposed that natural cross-linking might occur with the aging of corneal tissues, possibly leading to spontaneous stabilization of keratoconus with advanced age (11, 41). Age-related differences in keratoconus clinical findings are not clearly known, and longitudinal studies are necessary in the future.

The current study showed the distribution of pediatric keratoconus in a Chinese population in terms of gender and age groups, which may improve our understanding of disease onset and progression. However, several limitations must be noted. Firstly, this study was conducted in a single hospital. Although this hospital is a tertiary hospital with experienced ophthalmologists and a relatively large number of outpatient visits, the study does not fully represent the whole population of China. Secondly, the study evaluated the keratoconus clinical findings based on different gender and age groups. More detailed demographic information on atopy, eye rubbing, and sex hormones that affect pediatric keratoconus was limited in the current analysis. The present study provided references for improving the management of pediatric keratoconus to some extent, and further research should be conducted to overcome the above limitations.

## Conclusion

Males had a younger age and a higher ratio of advanced keratoconus than females in the pediatric keratoconus cases evaluated. The findings on age and gender-related differences provide assistance for clinicians in better understanding and managing pediatric keratoconus patients, and further investigation of this area is warranted.

## Data availability statement

The raw data supporting the conclusions of this article will be made available by the authors, without undue reservation.

## Ethics statement

The studies involving human participants were reviewed and approved by the Institutional Review Board of Henan Eye Hospital [ethical approval number: HNEECKY-2019 (5)]. Written informed consent to participate in this study was provided by the participants or their legal guardian/next of kin.

## Author contributions

KY: writing – original draft preparation, methodology, and software. YG: writing – original draft preparation, methodology, and writing – reviewing and editing. LX: methodology and writing – reviewing. QF: data curation and investigation. MZ: visualization and methodology. QW and SY: data curation. BZ: visualization and investigation. CP: software, validation, and supervision. SR: conceptualization and writing – reviewing and editing. All authors contributed to the article and approved the submitted version.

## Conflict of interest

The authors declare that the research was conducted in the absence of any commercial or financial relationships that could be construed as a potential conflict of interest.

## Publisher’s note

All claims expressed in this article are solely those of the authors and do not necessarily represent those of their affiliated organizations, or those of the publisher, the editors and the reviewers. Any product that may be evaluated in this article, or claim that may be made by its manufacturer, is not guaranteed or endorsed by the publisher.
